# Potential of micro-exercise to prevent long-term sickness absence in the general working population: prospective cohort study with register follow-up

**DOI:** 10.1038/s41598-022-06283-8

**Published:** 2022-02-10

**Authors:** Lars L. Andersen, Sebastian V. Skovlund, Jonas Vinstrup, Niels Geisle, Stig I. Sørensen, Sannie V. Thorsen, Emil Sundstrup

**Affiliations:** 1 National Research Centre for the Working Environment, Copenhagen, Denmark; 2 Sport Sciences, Department of Health Science and Technology, Aalborg University, Aalborg, Denmark; 3 Research Unit for Muscle Physiology and Biomechanics, Department of Sports Science and Clinical Biomechanics, University of Southern Denmark, Odense, Denmark; 4 The Danish Sector Working Environment Council - Welfare & Public Administration, Copenhagen, Denmark

**Keywords:** Environmental social sciences, Risk factors

## Abstract

This study assesses the potential of workplace-based micro-exercise (brief and simple exercise bouts) to prevent long-term sickness absence (LTSA) at the population level. In the Work Environment and Health in Denmark Study (2012–2018), we followed 70,130 workers from the general working population, without prior LTSA, for two years in the Danish Register for Evaluation of Marginalisation. We used Cox regression with model-assisted weights and controlled for various confounders. From 2012 to 2018, the percentage of workers in Denmark using workplace-based micro-exercise during and outside of working hours increased from 7.1 to 10.9% and from 0.8 to 1.4%, respectively. The incidence of long-term sickness absence (at least 30 days) was 8.4% during follow-up. The fully adjusted model showed reduced risk of long-term sickness absence from using micro-exercise during working hours, (HR 0.86, 95% CI 0.77–0.96), but not when used outside of working hours. If used by all workers, micro-exercise during working hours could potentially prevent 12.8% of incident long-term sickness absence cases (population attributable fraction). In conclusion, micro-exercise performed during working hours holds certain potential to prevent incident long-term sickness absence in the general working population. Large-scale implementation of workplace-based micro-exercise may represent an unexploited opportunity for public health promotion.

## Introduction

Sickness absence from work remains a major public health challenge with economic consequences for societies, employers and workers in terms of sickness benefit payments, lost productivity, lost earnings, and potential loss of paid employment^1,2^. Long-term sickness absence (LTSA) is especially problematic, accounting for up to 3/4 of total absence costs although constituting only a third of all lost working days^3^. In occupational research and practice, efforts to protect the health of workers have typically focused on reducing risk factors in the work environment, e.g. ergonomic and psychosocial risk factors^4^. By contrast, public health recommendations have largely focused on improving lifestyle, e.g. increasing physical activity and reducing sedentary behaviour^5^. However, during the last two decades, neither of these diverging efforts have succeeded in reducing sickness absence at the population level^2,6^. During recent years, occupational practice has integrated methods traditionally rooted in the public health domain, e.g. health promotion at the workplace in an attempt to prevent musculoskeletal disorders and sickness absence. While this may not be without challenges and remains far from widespread, randomized controlled trials (RCTs) assessing the effect of health promotion at the workplace have provided promising results in terms of workers’ somatic and mental health^7–9^. For example, some workplace interventions have used *micro-exercise*, i.e. simple and brief strengthening exercises designed to strengthen the primary muscles used during work^10,11^. Following this, micro-exercise can therefore be performed with elastic resistance bands together with colleagues at the local workstation; typically for 10 min three times a week without the need for changing clothes, going to a gym or showering afterwards. Micro-exercises may also be performed without exercise equipment (e.g. back exercises).

Systematic reviews of RCTs have found that brief bouts of workplace-based physical exercise are effective in improving somatic health symptoms like musculoskeletal pain among workers in sedentary and physically demanding jobs alike^7,8^. Indeed, individual RCTs demonstrate protective effects on work ability from engaging in 10–15 min of micro-exercise three times per week in job groups characterized by physically demanding work and short education^12–14^, i.e. groups of society who are typically difficult to reach with public health recommendations. These RCTs also show improvement of psychological and social factors from performing micro-exercise with colleagues at the workplace; including social climate, feelings of vitality and the ability to work together in teams^15,16^. By contrast, such effects were not observed for workers performing micro-exercise at home^12,15^, highlighting the importance of the social setting in which these are performed. Thus, the workplace constitutes a promising and not yet fully explored arena for improving public health.

However, despite positive results, challenges exist in the translation of findings from workplace RCTs to health and sickness absence at the population level. First, most RCTs have inadequate statistical power to assess the effect of workplace health promotion on sickness absence^17,18^. Second, such studies often rely on self-reported sickness absence as outcome, which is susceptible to recall and reporting bias. Third, dropout from interventions and loss to follow-up can hamper validity of the findings. Fourth, RCTs with a few hundred participants in selected companies are hardly representative of the general working population. Alternatively, large and well-designed prospective cohort studies drawing on representative samples of the general working population with loss-free follow-up from high-quality national registers, are ideal in the evaluation of workplace-based health promotion strategies aiming to reduce sickness absence. As it stands, Denmark is currently one of the few countries in the world where such national registers exist^19^. Based on the positive findings from smaller RCT’s we hypothesize that micro-exercise is prospectively associated with reduced risk of LTSA.

This study assesses the potential of workplace-based micro-exercise to prevent LTSA from work. The study uses representative data of the general working population in Denmark, without prior LTSA, combined with high-quality national registers (used for follow-up).

## Methods

### Study design and population

This prospective study uses the 2012, 2014, 2016 and 2018 rounds of the Work Environment and Health in Denmark Study (WEHD)^20^ combined with the Danish Register for Evaluation of Marginalisation (DREAM)^21^. The databases were merged on the secure server of Statistics Denmark using the unique personal identification number (CPR) given to all Danish citizens at birth or immigration.

Prior to each round of WEHD, Statistics Denmark drew probability samples of wage earners aged 18–64 years, with an income of at least 3000 DKK (400 €) per month during the past 3 months and having paid employment for at least 35 h per month. Invitations were sent to 228,173 individuals of which 127,882 (56%) responded. We included only individuals who confirmed on the questionnaire to be currently employed wage earners (n = 110,357). For individuals participating in more than one round of WEHD, we included only first occasion responses (n = 73,298). Finally, we included only wage earners free from LTSA during the 52 weeks preceding baseline and those replying to the questions about micro-exercise (n = 70,130). Reporting follows the STROBE guidelines (for prospective cohort studies)^22^.

### Micro-exercise (predictor)

The questions about offer and use, respectively, of workplace-based micro-exercise built on a questionnaire matrix from the Danish Working Environment Cohort Study from 2010^23^. Participants were asked, ‘During the last year, have you been offered the following health promotion through your workplace?’ For the category of micro-exercise, the phrasing was ‘Small exercise activities during the day (e.g. elastic band exercises, back exercises)’, where participants could reply the following: (1) No, (2) Yes, offered during working hours, (3) Yes, offered outside of working hours. For those replying yes (last two options), an additional question was posed ‘Did you use the offer?’, with response options (1) No, (2) Yes.

Subsequently, we calculated ‘uptake’ as the percentage of workers replying ‘yes’ to using micro-exercise among those who were offered the micro-exercise.

### Long-term sickness absence (outcome)

The DREAM register holds high-quality weekly information about sickness benefit reimbursement from the municipalities to the employer. The first 30 days of sickness absence are financially covered by the employer, after which the municipality can reimburse the remaining days. The validity of this register is high as the employer has a strong economic incentive to receive the reimbursement^21^. LTSA was defined as having registered sickness absence for at least 30 days for a period of up to 2 years, starting the week after the questionnaire reply. DREAM contains weekly—and not daily—information about reimbursement of sickness absence payments. Thus, at least 30 days of consecutive sickness absence corresponds to 6 consecutive weekly registrations in DREAM as the first week of sickness absence may begin on the last day of the week, and the last week of sickness absence may begin on the first day of the week (i.e. 1 + 4 × 7 + 1 days = 30 days). For the last round (2018) of the questionnaire survey, the follow-up period is limited to the most recent update of the DREAM register (about 1½ years follow-up time).

### Control variables

Age (continuous variable) and sex (man, woman) for each participant were drawn from the Central Person Register of Denmark. Highest completed education (unskilled, skilled and further education) and occupation (1st level ISCO codes) were also drawn from registers. Survey year (2012, 2014, 2016, 2018) was entered as a categorical variable. The psychosocial work factors were based on the Copenhagen Psychosocial Questionnaire (COPSOQ) and included scales of influence at work (two items) and work-life balance (six items) that each was converted to a scale of 0–100^24^, and included as continuous variables. Lifestyle included leisure-time physical activity levels (continuous variable, total weekly hours of leisure physical activity), smoking (categorical variable: daily, once in a while, ex-smoker, never), and body mass index (continuous variable, BMI, kg m^−2^). For health-related variables, depressive symptoms (Major Depression Inventory, scale 0–50) were included as a continuous variable, and frequency of pain during the last three months (daily, weekly, monthly, a few times, not at all) was included as a categorical variable.

### Statistical analyses

The development in Denmark from 2012 to 2018 in (1) offer, (2) use and (3) uptake of micro-exercise during and outside of working hours, respectively, were described with weighted frequencies (Proc Surveyfreq of SAS version 9.4). Using model-assisted weights based on high-quality national registers, each respondent was assigned a weight variable to ensure that the estimates were representative of the general working population in Denmark.

Using the Cox proportional hazard model with weights (Proc SurveyPhreg of SAS version 9.4.), we modelled the hazard ratio (HR) of LTSA (outcome) as a time-to-first event analysis from the use of micro-exercise (predictor variable) during and outside of working hours (reference: no use). We censored in case of reaching the end of follow-up, early retirement, disability pension, statutory retirement, emigration, or death. Missing data were not imputed as the weight variable repairs both non-response and deviations of the probability sample from the general working population. Model 1 (minimally adjusted) was adjusted for age, sex, education, and year of questionnaire reply. Model 2 (fully adjusted) additionally controlled for psychosocial work factors, lifestyle, depressive symptoms, and pain frequency. Model 3 was a sensitivity analysis of the fully adjusted model including only individuals free from mental and somatic health problems at baseline (normal score on depressive symptoms and monthly or less frequent pain) to investigate the potential effect of micro-exercise for preventing LTSA in a completely healthy population. Results are reported as HRs and 95% confidence intervals (95% CI). Finally, we tested whether use of micro-exercise interacted with age, sex and education, respectively, regarding the risk of LTSA.

The population attributable fraction (PAF), which expresses the contribution of a risk factor to LTSA, was calculated based on the HRs and proportions exposed (Pe) from the fully adjusted model, but redefining the reference group to those using micro-exercise during working hours. Using this reverse procedure, we calculated risk estimates for those not using micro-exercise during working hours (i.e. not using and only using outside of working hours). PAF (%) was calculated as ∑Pe(HRe − 1)/(∑ Pe(HRe − 1) + 1) × 100%.

### Ethical approval

According to Danish law, questionnaire and register-based studies do not need approval by an ethical committee nor informed consent (https://www.nvk.dk/forsker/naar-du-anmelder/hvilke-projekter-skal-jeg-anmelde).

## Results

Table 1 shows the descriptive baseline characteristics of the 70,130 included participants.

**Table 1:** Unweighted descriptive characteristics of the participants (N = 70,130) at baseline.

	N	%	Mean	SD
**Questionnaire round**				
2012	19,709	28.1		
2014	15,070	21.5		
2016	18,228	26.0		
2018	17,123	24.4		
**Age (years)**	70,130		45.9	10.8
**Sex**				
Men	33,292	47.5		
Women	36,838	52.5		
**Highest education attained**				
Less than vocational or vocational education	38,092	54.7		
Higher education	31,591	45.3		
**BMI (kg m**^**−2**^**)**	67,982		25.7	4.4
**Physical activity during leisure (hours per week)**	68,394		5.2	3.3
**Smoking**				
Yes, daily	9943	14.5		
Yes, once in a while	3554	5.2		
Ex-smoker	19,797	29.0		
No, never	35,090	51.3		
**Psychosocial work factors (0–100)**				
Work-life balance	69,884		46.2	16.3
Influence at work	70,072		78.8	19.0
**Pain frequency during the last 3 months**				
Daily	10,485	15.3		
Weekly	12,067	17.6		
Monthly	9667	14.1		
Seldomly	21,095	30.7		
Never	15,440	22.5		
**Major Depression Inventory (0–50)**	68,497		8.1	7.3

Values are percentage of participants or mean and standard deviations (SD).

**Figure 1:**
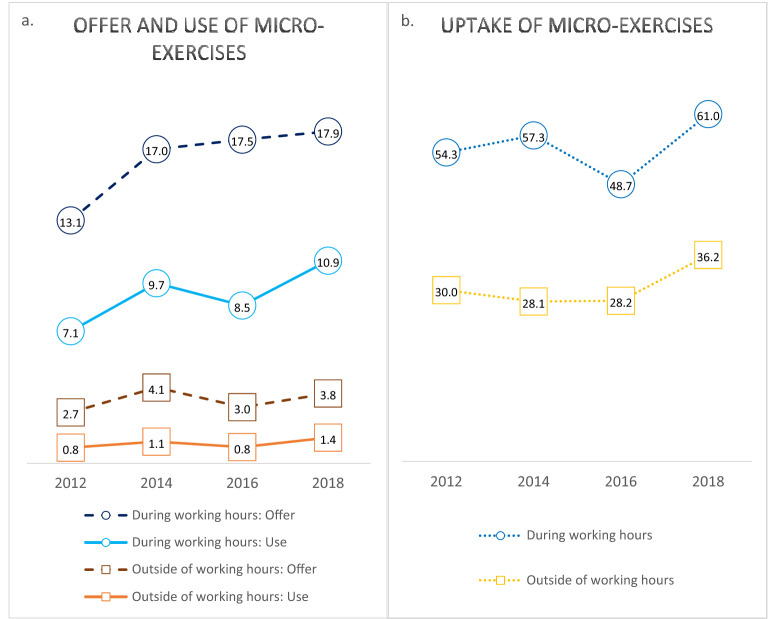
*Left *(**a**) Offer (broken lines) and use (full lines) of micro-exercise during (circles) and outside (squares) of working hours from 2012 to 2018 in the general working population without LTSA at baseline. *Right *(**b**) Uptake of micro-exercise during and outside of working hours (percentage users among those offered). All numbers of weighted percentages representative of the general working population in Denmark without prior long-term sickness absence.

**Figure 2:**
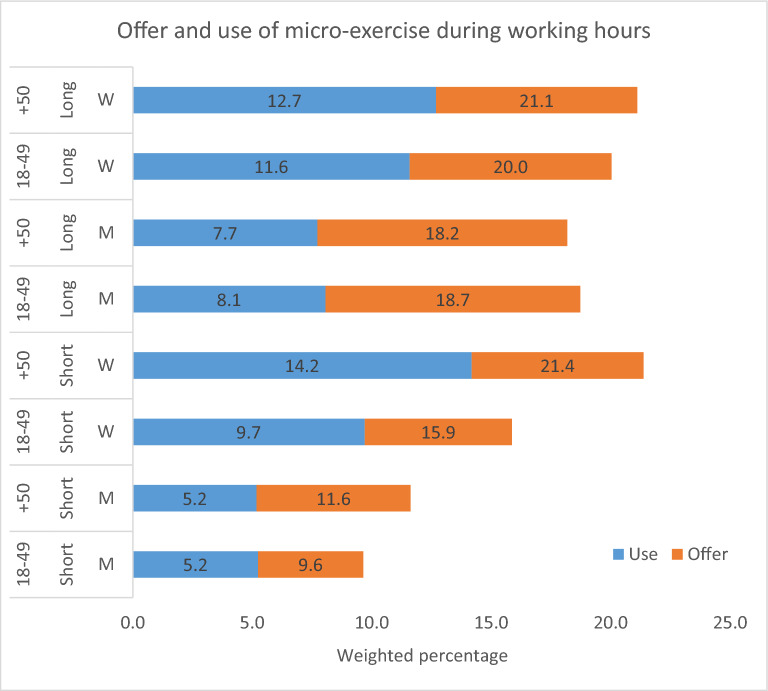
Offer and use of micro-exercise during working hours (weighted percentages) across different strata of education (Short = unskilled and skilled, Long = further education), sex (W = women, M = men) and age (18–49, + 50 years). Data pooled for the years 2012, 2014, 2016 and 2018.

Figure 1a shows that from 2012 to 2018, the percentage of workers being *offered* micro-exercise increased from 13.1 to 17.9% during working hours and from 2.7 to 3.8% outside of working hours. In the same period the percentage of workers *using* micro-exercise increased from 7.1 to 10.9% during working hours and from 0.8 to 1.4% outside of working hours. Figure 1b shows that the *uptake* of micro-exercise among those offered the opportunity increased from 54.3 to 61.0% during working hours and from 28.1 to 36.2% outside of working hours. Figure 2 shows *offer* and *use* of micro-exercise during working hours for all four rounds combined, stratified by education, sex and age. Offer and use of micro-exercise during working hours were generally lower among men than women, especially among men with short- or no education.

In the general working population without prior LTSA, the weighted incidence of LTSA during follow-up was 8.4%. The weighted mean follow-up time until an event of LTSA or until censoring was 94.7 weeks, corresponding to a total of 127,718 person-years. Table 2 presents the minimally and fully adjusted models, as well as the sensitivity analysis, for the risk of LTSA. The fully adjusted model showed reduced risk of LTSA from using micro-exercise during working hours (HR 0.86, 95% CI 0.77–0.96), but not when used outside of working hours (HR 0.90, 95% CI 0.67–1.20) compared with reference (i.e. not using micro-exercise). Consistent results were found in the the sensitivity analysis (Model 3) including only individuals completely healthy at baseline. The use of micro-exercise did not interact with age, sex or education for the risk of LTSA. PAF analyses of the fully adjusted model show that 12.8% of incident LTSA cases could be attributable to the absence of performing micro-exercise during working hours.

**Table 2:** Hazard ratios and 95% confidence intervals for the risk of long-term sickness absence during follow-up from use of micro-exercise during and outside of working hours.

Using micro-exercise	N	Weighted percentage	HR (95% CI)		
			Model 1	Model 2	Model 3
No	62,652	90.2%	1	1	1
During working hours	6750	8.8%	**0.80 (0.71–0.89)**	**0.86 (0.77–0.96)**	**0.81 (0.68–0.95)**
Outside working hours	728	1.0%	0.91 (0.68–1.21)	0.90 (0.67–1.20)	1.02 (0.67–1.57)

Significant findings are marked in bold.

Model 1: Controlled for age, sex, education, survey year.

Model 2: Controlled for age, sex, education, survey year, lifestyle (BMI, smoking, leisure physical activity), psychosocial work factors (work-life balance and influence at work), depressive symptoms (MDI), and pain frequency.

Model 3: Sensitivity analysis of model 2, including only healthy individuals at baseline (normal MDI score, monthly or less frequent pain)(n = 45,570).

## Discussion

The present study shows that micro-exercise performed during working hours holds certain potential to prevent LTSA in the general working population. Importantly, the use of micro-exercise did not interact with age, sex and education in the risk of LTSA, indicating a potential for prevention of LTSA across the entire general working population. Although the percentage of workers in Denmark who were offered and used micro-exercise increased from 2012 to 2018, both offer and use hereof is still rather limited at the population level, emphasizing that a possible unexploited potential exists, especially among men and workers with short- or no education.

This prospective cohort study including more than 70,000 workers and register-based follow-up elaborates on previous findings from small-scale RCTs with self-reported outcomes, showing that micro-exercise performed during working hours has the potential to prevent health deterioration. However, the majority of RCTs have had inadequate statistical power to assess the effect of micro-exercise on sickness absence or used self-reported sickness absence as outcome^17^. In the present study, micro-exercise performed outside of working hours was—in contrast to micro-exercise performed during working hours—not associated with reduced risk of LTSA. This is in line with RCT’s showing that micro-exercise performed at the workplace exhibit the potential to increase muscle strength, reduce musculoskeletal pain, prevent deterioration of work ability and improve psychosocial work factors, whereas micro-exercise performed at home yield no such benefits^10,12,15^. An important factor is the lower adherence to home versus workplace micro-exercise, which per se provides a lower physiological training stimulus^10^. In the present study, the higher uptake among those offered micro-exercise during versus outside of working hours further supports this. However, underneath this notion lies important psychosocial aspects; both in terms of using the social setting at the workplace to improve uptake and adherence, but also in the acknowledgement of the fact that overall health includes a plurality of psychological and social aspects^25^. As the general working population spend a large part of their life at work, the workplace represent an ideal arena for health promotion, where workers who are unable to find the time and motivation to do regular physical exercise during leisure time can perform micro-exercise together with their colleagues at the workplace instead. Thus, the underlying mechanisms of LTSA prevention from micro-exercise during working hours are likely to be multifactorial in nature, including both physiological, psychological and social factors.

In contrast to aforementioned studies and as is evident from the applied methodology, the present study sought to shed light on “the bigger picture”; i.e. answering the question of *whether* micro-exercise during and outside of working hours could potentially prevent incident LTSA in the general working population. Therefore, the present study did not investigate *how* to increase the offer and use of micro-exercise in the general working population. The offer of micro-exercise at the workplace may be dependent on the willingness of companies to allow workers to engage in physical exercise during working hours. However, as the employer is responsible for covering financial expenses during the initial 30 days of sickness absence, the potential of micro-exercise to prevent health deterioration represents a strong economic incentive for successful implementation at the workplace. Additionally, the present study showed that micro-exercise performed during working hours could—if used by all workers—potentially prevent 12.8% of all new LTSA cases in the general working population. From the workers' perspective, uptake of micro-exercise and long-term adherence may depend on several factors, including perceived relevance, motivation, and actual possibilities to utilize the local work environment as an arena for health promotion. For example, process evaluations of small-scale intervention studies have identified several barriers to engaging in physical activity during working hours, with lack of time being the most consistently reported barrier^26^. In the attempt to counteract this, most common facilitators include information meetings, kick-off events, and local ambassadors to encourage implementation of physical activity at the workplace^26^. Thus, workplaces offering micro-exercise to its workers would likely benefit from allocating sufficient time and resources to allow for the successful implementation of 10–15 min during the work day to perform such exercises. Above the company and worker level, a national approach with targeted health promotion strategies may also constitute a viable future course of action. For example, at the population level, national campaigns from different countries have led to more positive beliefs about physical activity in relation to musculoskeletal pain^27–30^, although with minor impact on sickness absence. In Denmark, the national Job & Body campaign from 2011 to 2014 may have contributed to the increase in offer and use of micro-exercise seen from 2012 to 2018^27^: During this campaign, researchers and local practioners sought to spread the advice of (1) staying active even in periods of musculoskeletal pain, (2) to focus on prevention and not only rehabilitation of musculoskeletal pain, (3) to create a balance between individual demands of the job and the capacity of the body, (4) to perform micro-exercise with colleagues at the workplace, and (5) to highlight and manage physical wellbeing together at the workplace. Based on feedback from workplaces, micro-exercise with elastic bands was identified as the most practically useful part of the campaign; providing a potential direction for future implementation of workplace physical activity. Still, with only a tenth of the general working population engaging in micro-exercise during working hours in 2018, future strategies seeking to encorporate physical activity at the workplace holds great potential.

### Limitations and strengths

A limitation of Danish registers is that, due to privacy matters, there is no registration of the reason or specific disease underlying the LTSA. LTSA is therefore an unspecific proxy for poor health, although a proxy strongly related to serious health endpoints, such as disability and mortality^1^. Even though we controlled the analyses for several possible confounders, including education, occupation, lifestyle, psychosocial work factors, pain frequency and depressive symptoms, it can be argued that the true preventive potential of an intervention can only be investigated in a healthy population. Therefore, we excluded those with LTSA prior to baseline. Furthermore, we performed a sensitivity analysis including only individuals free from mental and somatic health problems at baseline, i.e. with a normal depressive symptoms score and with little or no pain. The sensitivity analysis confirmed the main analyses, which strengthens the validity of the findings. Unmeasured or residual confounding may exist. However, as we already controlled the analyses for various confounders this is unlikely to markedly influence the main findings. Lastly, the use of micro-exercise did not interact with age, sex or education for the risk of LTSA, suggesting that the potential for prevention is generalizable across different groups of society. Based on cross-country comparisons of work environment and health, Denmark is close to the average of the European Union^31^, suggesting that the results are not only generalizable to the general working population of Denmark, but possibly also to a wider audience of countries, although this should be investigated in more detail.

## Conclusion

In conclusion, engaging in micro-exercise during working hours holds certain potential to prevent incident long-term sickness absence in the general working population. Although the use of such exercises has increased from 2012 to 2018, a possible opportunity for public health promotion at the workplace remains unexploited.

## Data Availability

The authors encourage collaboration and use of the data by other researchers. Data is stored on the secure server of Statistics Denmark, and researchers interested in using the data for scientific purposes should contact the corresponding author.
